# Supplementary material for: Multi-omics characterization of autophagy-related molecular features for therapeutic targeting of autophagy
Source: Nat Commun. 2022 Oct 26;13:6345. doi: 10.1038/s41467-022-33946-x (PMC9606020; doi:10.1038/s41467-022-33946-x)

Supplementary Information

Multi-omics characterization of autophagy-related molecular features for therapeutic targeting of autophagy

Mei Luo[1,2,3,#], Lin Ye[1,#], Ruimin Chang[1,#], Youqiong Ye[3,4], Zhao Zhang[3], Chunjie Liu[2,3], Shengli Li[3], Ying Jing[3], Hang Ruan[3], Guanxiong Zhang[1], Yi He[1], Yaoming Liu[3], Yu Xue[2], Xiang Chen[1,*], An-Yuan Guo[2,*], Hong Liu[1,*], Leng Han[3,5,6,*]

Inventory of Supplementary Information

Supplementary Fig. 1 Confounding factors between autophagy score-high and autophagy score-low groups.

Supplementary Fig. 2 Expression of significantly altered mRNAs and their associations with drugs.

Supplementary Fig. 3 Regulatory networks between miRNAs, mRNAs and TFs.

Supplementary Fig. 4 Landscape of drug response across multiple cancer types.

Supplementary Fig. 5 Characterization of autophagy sensitizing drug response *in vitro* and *in vivo*.

Supplementary Fig. 6 Analysis of autophagy sensitizing drug response.

Supplementary Fig. 7 Expression of DDIT4 and the efficiency and specificity of DDIT4 siRNAs or DDIT4-OE lentivirus and ATG5 siRNAs in vitro.

Supplementary Fig. 8 Uncropped scans of all blots.

Supplementary Fig. 1

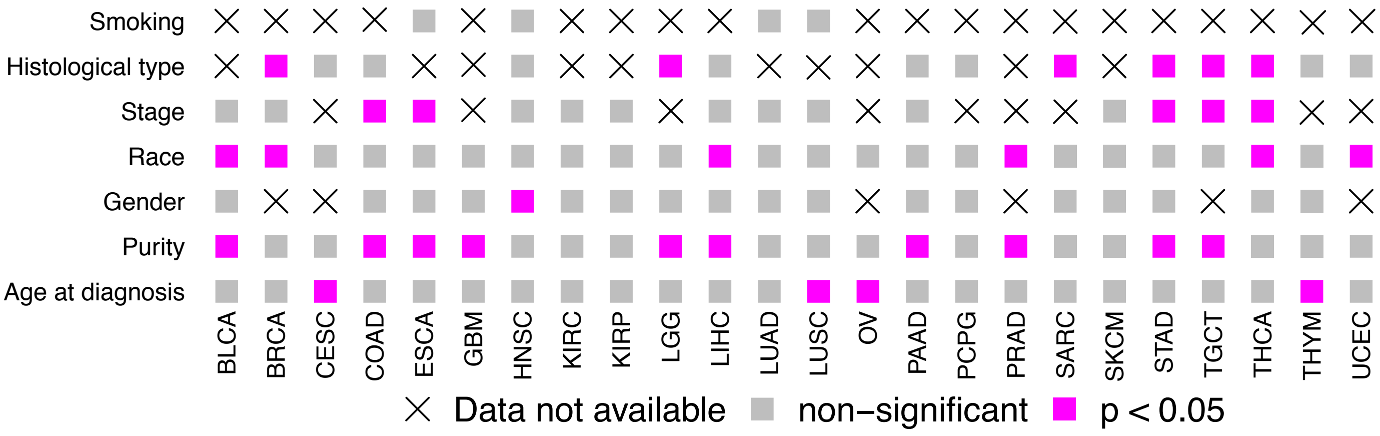

Supplementary Fig. 1 Confounding factors between autophagy score-high and autophagy score-low groups. Potential confounding factors across 24 cancer types. Heatmap shows p-values (two-sided t-test for continuous variables, including age at diagnosis and tumor purity, and chi-squared test for discrete variables, including gender ,race, pathologic stage, histological type, and smoking history) with significance ($p < 0.05$, red). Features marked as X indicate data not available. The exact p values are provided in Source Data file.

Supplementary Fig. 2

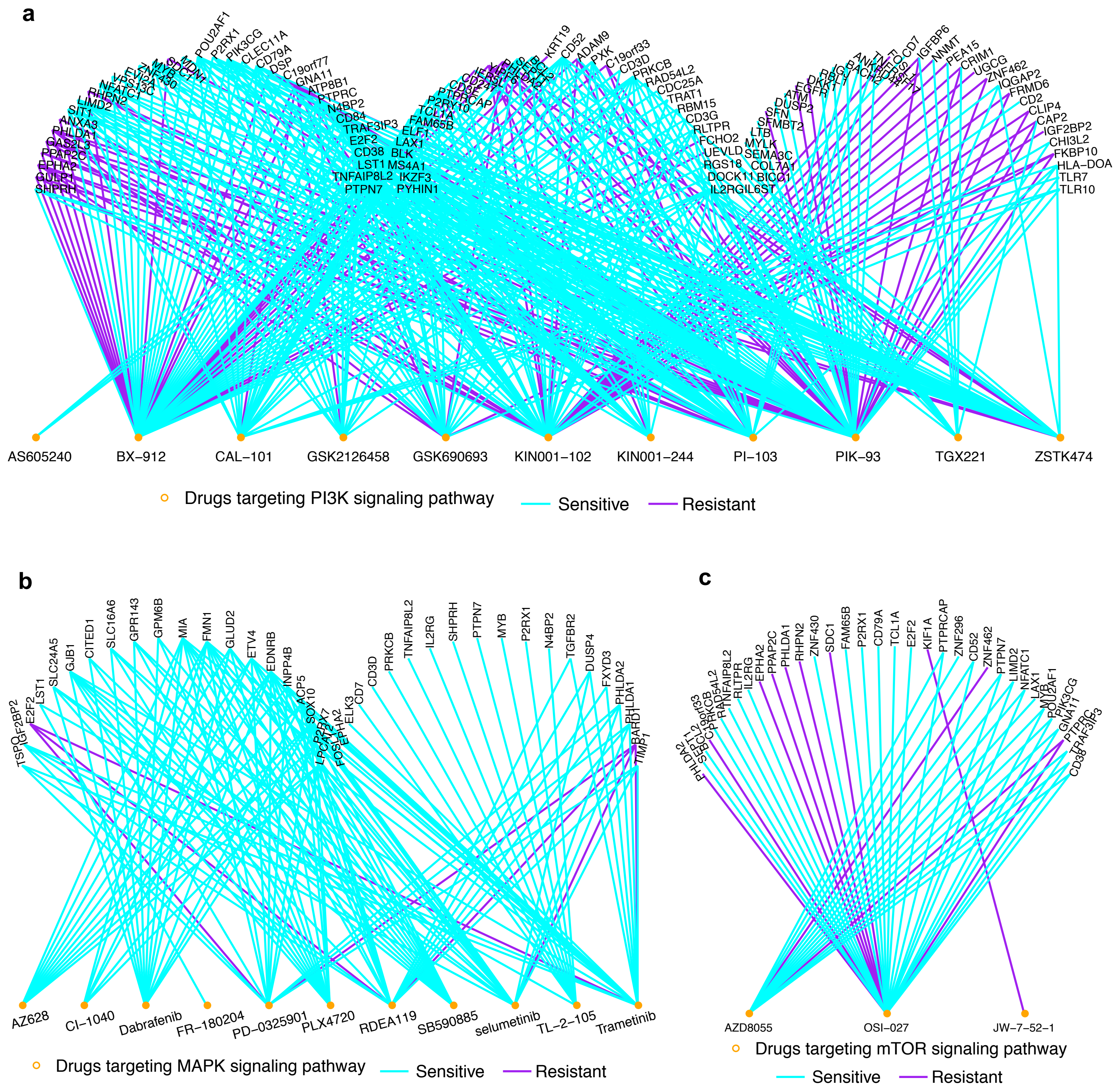

Supplementary Fig. 2 Expression of significantly altered mRNAs and their associations with drugs. (a) Associations between anti-cancer drugs from GDSC and expression of genes in PI3K signaling pathway. Orange dots denote drugs; cyan lines indicate sensitivity; purple lines indicate resistance. (b) Associations between anti-cancer drugs from GDSC and expression of genes in MAPK signaling pathway. Orange dots denote drugs; cyan lines indicate sensitivity ; purple lines indicate resistance. (c) Associations between anti-cancer drugs from GDSC and expression of genes in mTOR signaling pathway. Orange dots denote drugs; cyan lines indicate sensitivity; purple lines indicate resistance.

Supplementary Fig. 3

a

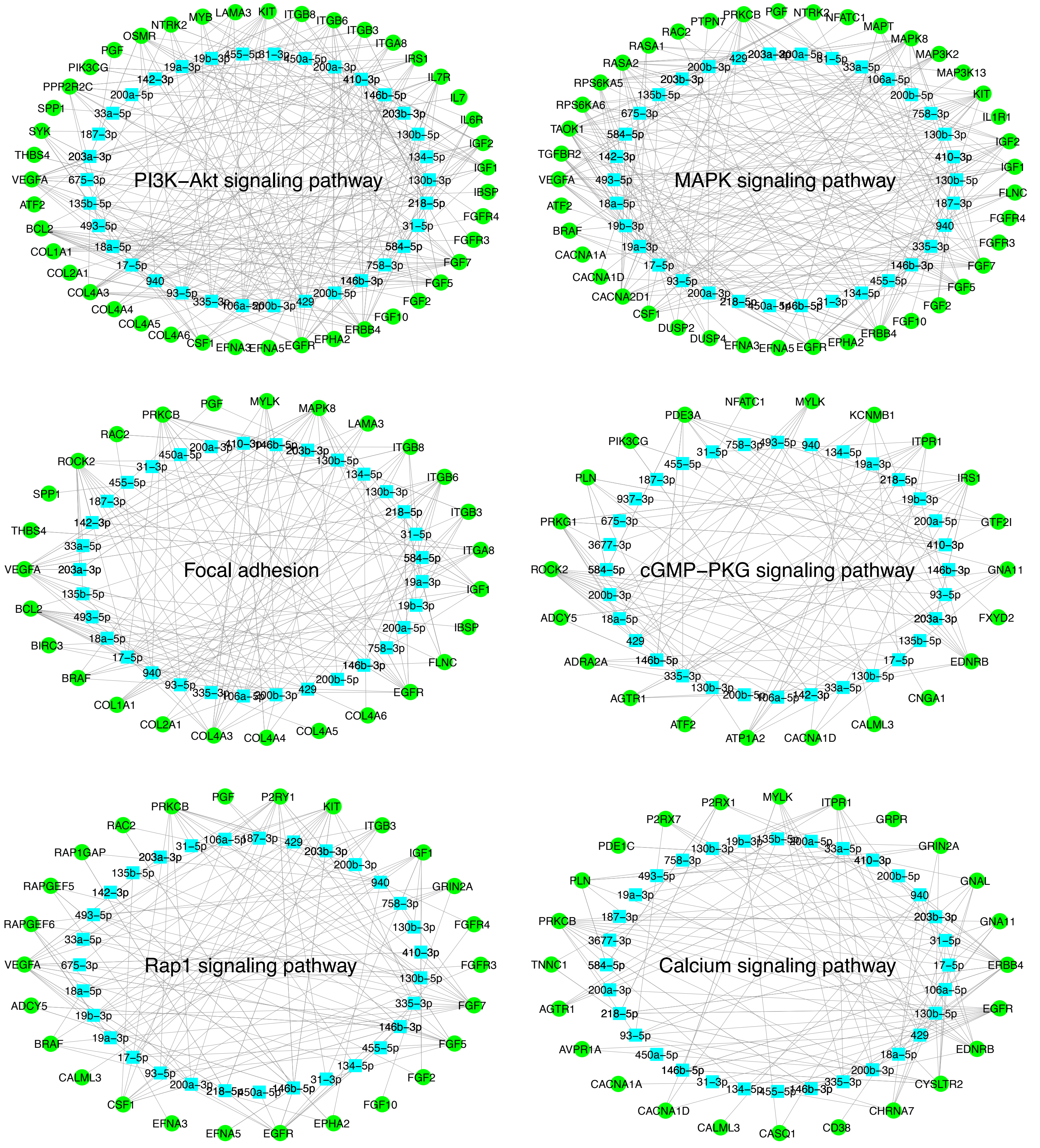

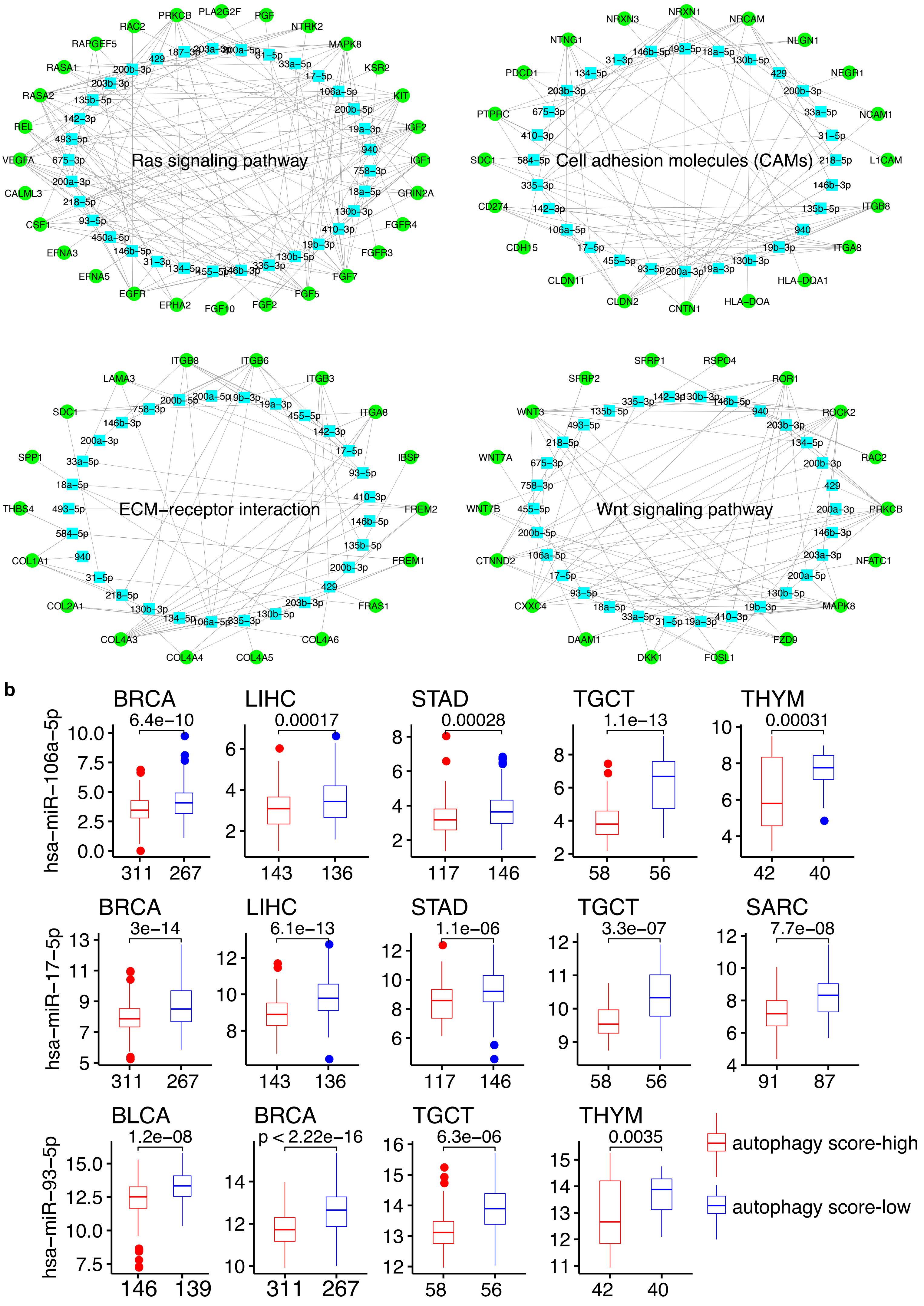
Ras signaling pathway
PRKCB
PLA2G2F
PGF
RAC2
NTRK2
RAPGEF5
MAPK8
RASA1
KSR2
RASA2
KIT
REL
IGF2
VEGFA
IGF1
CALML3
GRIN2A
CSF1
FGFR4
EFNA3
FGFR3
EFNA5
FGF7
EGFR
FGF5
EPHA2
FGF10
FGF2
Cell adhesion molecules (CAMs)
NRXN3
NRXN1
NRCAM
NTNG1
NLGN1
PDCD1
NEGR1
PTPRC
NCAM1
SDC1
L1CAM
CD274
ITGB8
CDH15
ITGA8
CLDN11
HLA-DQA1
CLDN2
HLA-DOA
CNTN1
ECM-receptor interaction
ITGB8
ITGB6
LAMA3
ITGB3
SDC1
ITGA8
SPP1
IBSP
THBS4
FREM2
COL1A1
FREM1
COL2A1
FRAS1
COL4A3
COL4A6
COL4A4
COL4A5
Wnt signaling pathway
SFRP1
RSPO4
SFRP2
ROR1
WNT3
ROCK2
WNT7A
RAC2
WNT7B
PRKCB
CTNND2
NFATC1
CXXC4
MAPK8
DAAM1
FZD9
DKK1
FOSL1
b
hsa-miR-106a-5p
BRCA 6.4e-10 311 267
LIHC 0.00017 143 136
STAD 0.00028 117 146
TGCT 1.1e-13 58 56
THYM 0.00031 42 40
hsa-miR-17-5p
BRCA 3e-14 311 267
LIHC 6.1e-13 143 136
STAD 1.1e-06 117 146
TGCT 3.3e-07 58 56
SARC 7.7e-08 91 87
hsa-miR-93-5p
BLCA 1.2e-08 146 139
BRCA p < 2.22e-16 311 267
TGCT 6.3e-06 58 56
THYM 0.0035 42 40
autophagy score-high
autophagy score-low

c

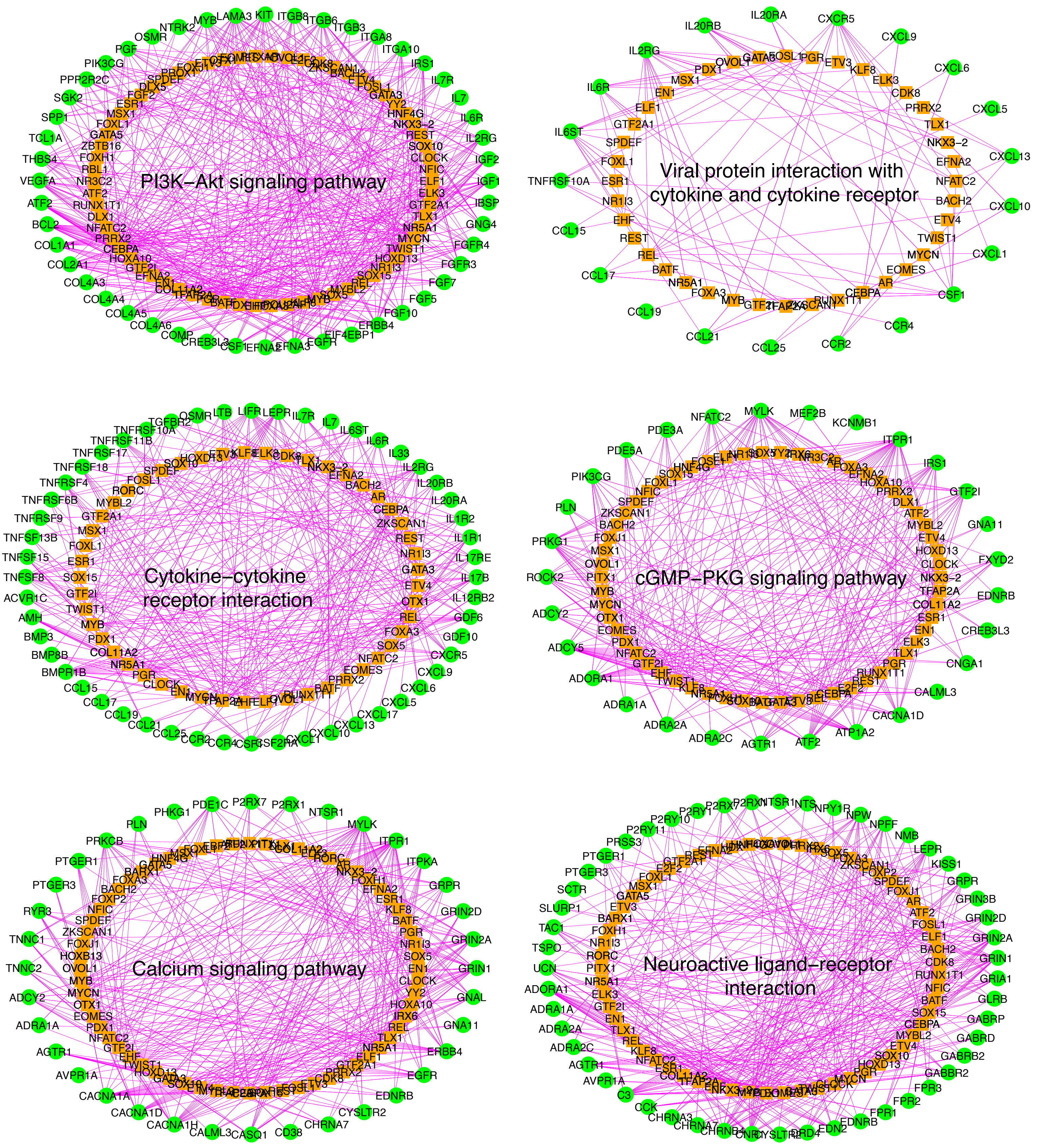
PI3K–Akt signaling pathway
Viral protein interaction with cytokine and cytokine receptor
Cytokine–cytokine receptor interaction
cGMP–PKG signaling pathway
Calcium signaling pathway
Neuroactive ligand–receptor interaction

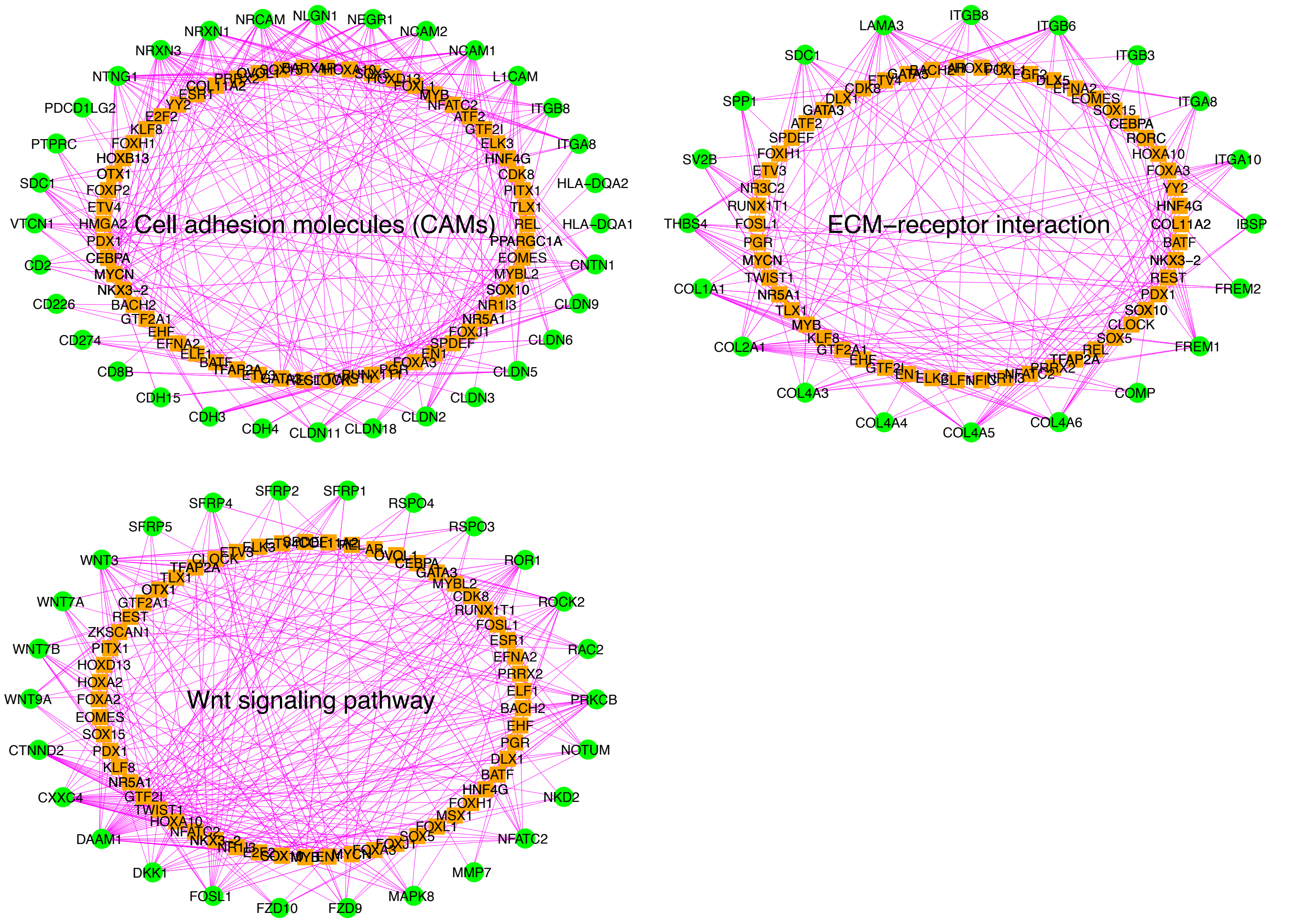

Supplementary Fig. 3 Regulatory networks between miRNAs, mRNAs and TFs. (a) miRNA-target regulatory networks of 10 significantly enriched KEGG pathways. Cyan rectangles denote miRNAs; green dots denote genes; cyan rectangle width shows number of cancer types with altered miRNA. (b) miRNA expression of miR 106a 5p, miR 17 5p, and miR 93 5p across multiple cancers. Red denotes autophagy score-high group; blue denotes autophagy score-low group; The x-axis denotes the number of samples. Two-sided student’ s t test was used for estimation of difference in two groups. The boxes show the median ± 1 quartile, with whiskers extending to the most extreme data point within 1.5 interquartile range from the box boundaries. (c) TF-target regulatory networks of 9 significantly enriched KEGG pathways. Orange rectangles denote TFs; green dots denote genes.

Supplementary Fig. 4

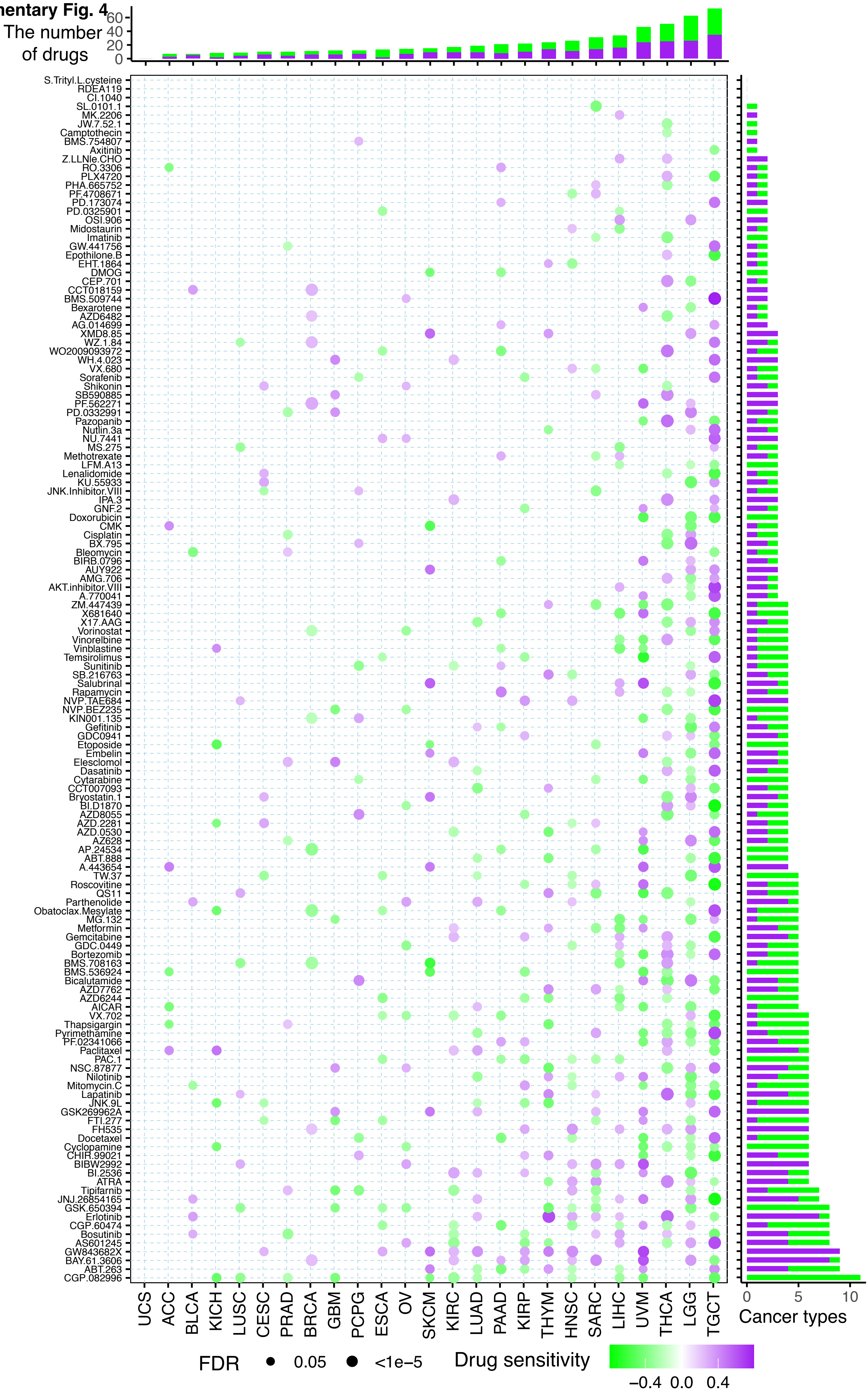

Supplementary Fig. 4 Landscape of drug response across multiple cancer types. Spearman correlation between autophagy scores and imputed data for response of TCGA tumor samples to 138 drugs. Purple dots denote a positive correlation (drug resistance); green dots denote a negative correlation (drug sensitivity). The Benjamini & Hochberg method was used for multiple hypothesis testing.

Supplementary Fig. 5

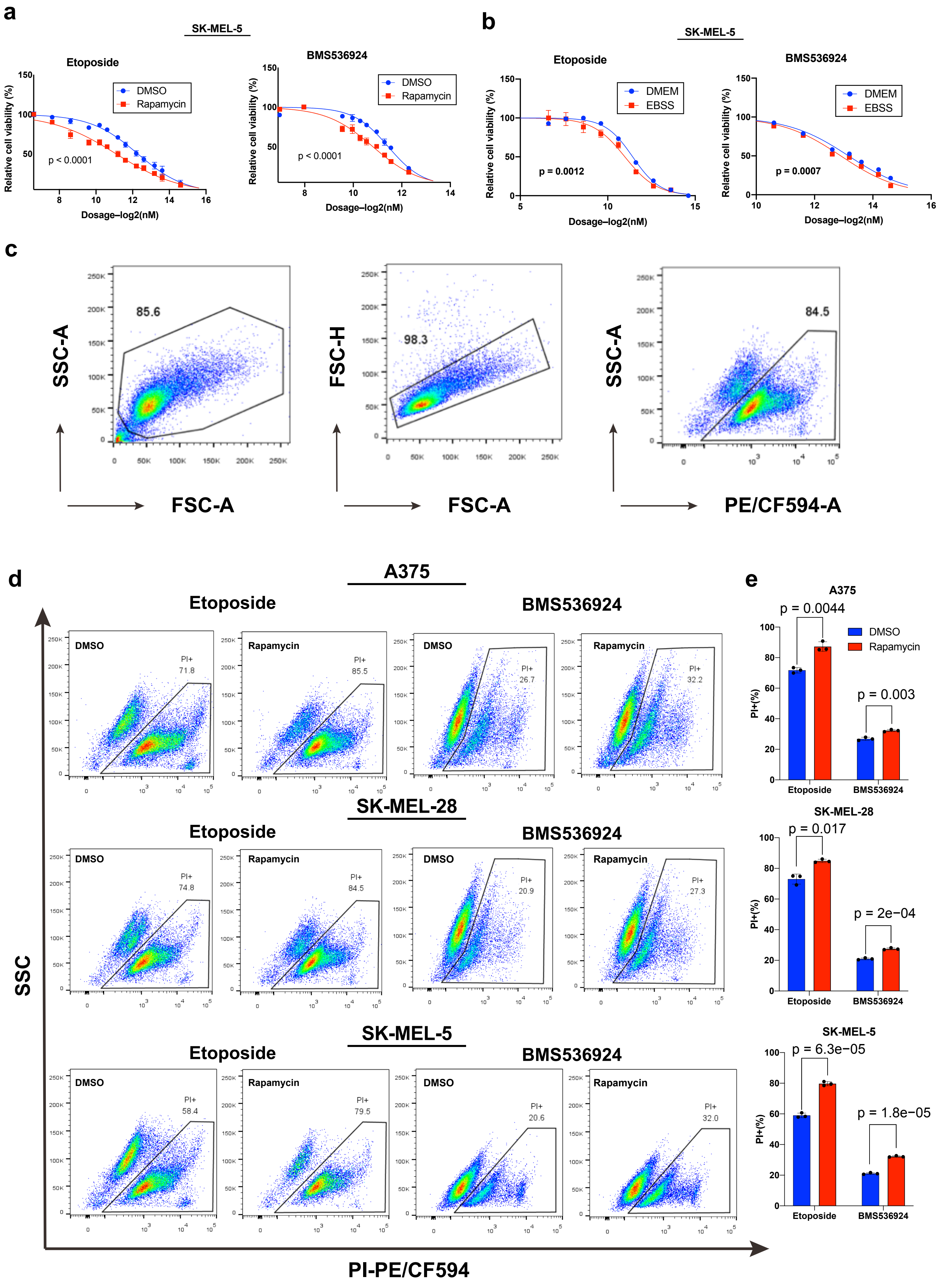

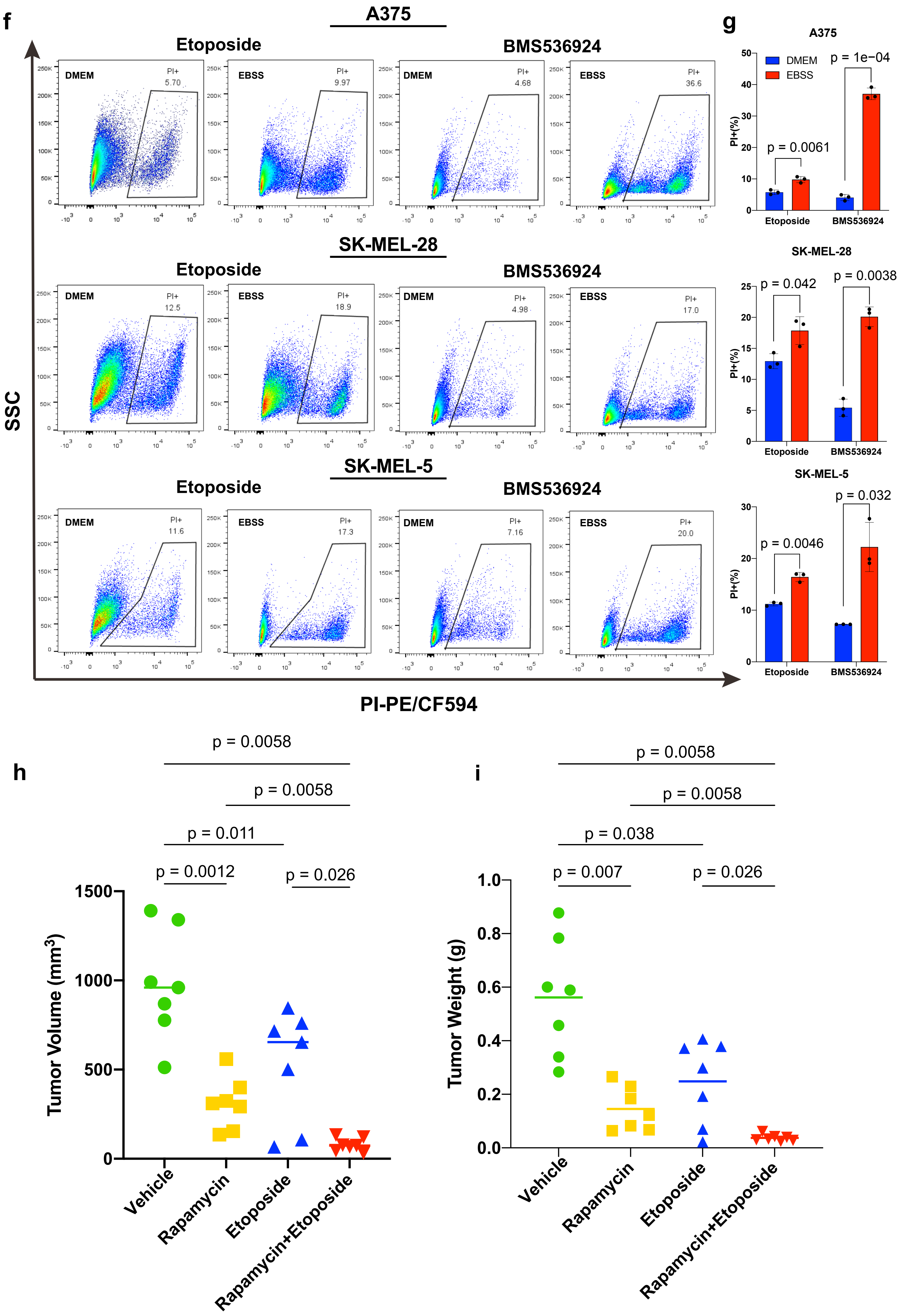
f
A375
Etoposide
BMS536924
DMEM
PI+
5.70
EBSS
PI+
9.97
DMEM
PI+
4.68
EBSS
PI+
36.6
SK-MEL-28
Etoposide
BMS536924
DMEM
PI+
12.5
EBSS
PI+
18.9
DMEM
PI+
4.98
EBSS
PI+
17.0
SK-MEL-5
Etoposide
BMS536924
DMEM
PI+
11.6
EBSS
PI+
17.3
DMEM
PI+
7.16
EBSS
PI+
20.0
SSC
PI-PE/CF594
g
A375
DMEM
EBSS
p = 1e−04
p = 0.0061
PI+(%)
Etoposide
BMS536924
SK-MEL-28
p = 0.042
p = 0.0038
PI+(%)
Etoposide
BMS536924
SK-MEL-5
p = 0.032
p = 0.0046
PI+(%)
Etoposide
BMS536924
h
p = 0.0058
p = 0.0058
p = 0.011
p = 0.0012
p = 0.026
Tumor Volume (mm3)
Vehicle
Rapamycin
Etoposide
Rapamycin+Etoposide
i
p = 0.0058
p = 0.0058
p = 0.038
p = 0.007
p = 0.026
Tumor Weight (g)
Vehicle
Rapamycin
Etoposide
Rapamycin+Etoposide

Supplementary Fig. 5 Characterization of autophagy sensitizing drug response in vitro and in vivo. (a) Dose-response curves for the mean value of cell viability of etoposide and BMS536924 in rapamycin-induced and non-induced conditions in the melanoma cell line SK-MEL-5. Cell viability was normalized to the level of cells treated with DMSO. Error bars indicate the mean ± SD. The drug screen data of different groups (n = 4) were fitted and compared by sigmoidal dose-response curves. (b) Dose-response curves for the mean value of cell viability of etoposide and BMS536924 in starvation-induced and non-induced conditions in the melanoma cell line SK-MEL-5. DMEM: Dulbecco's Modified Eagle Medium for control. EBSS: Earle's Balanced Salt Solution for starvation. Cell viability was normalized to the level of cells treated with DMSO. Error bars indicate the mean ± SD. The drug screen data of different groups (n = 4) were fitted and compared by sigmoidal dose-response curves. (c) Gating strategies for flow cytometric analysis of melanoma cells for propidium iodide (PI) expression. Cell death was quantified by the percentage of PI positive staining cells detected by flow cytometric analysis. (d & f) Representative cell death plots of melanoma cell lines (A375, SK-MEL-28, SK-MEL-5) treated with etoposide and BMS536924 in rapamycin-induced (d) and starvation-induced (f), and non-induced conditions. (e & g) Statistical data of the cell death in the (d , f) groups (n = 3) were analyzed by two-sided Student's t test. Data were presented as mean ± SD. (h & i) The tumor volume (h) or tumor weight (i) of mice as a function of time (n = 7/group). The difference in multiple groups was estimated by one-way ANOVA analysis. *P < 0.05; **P < 0.01; ***P < 0.001; ****P < 0.0001; ns is not significant.

Supplementary Fig. 6

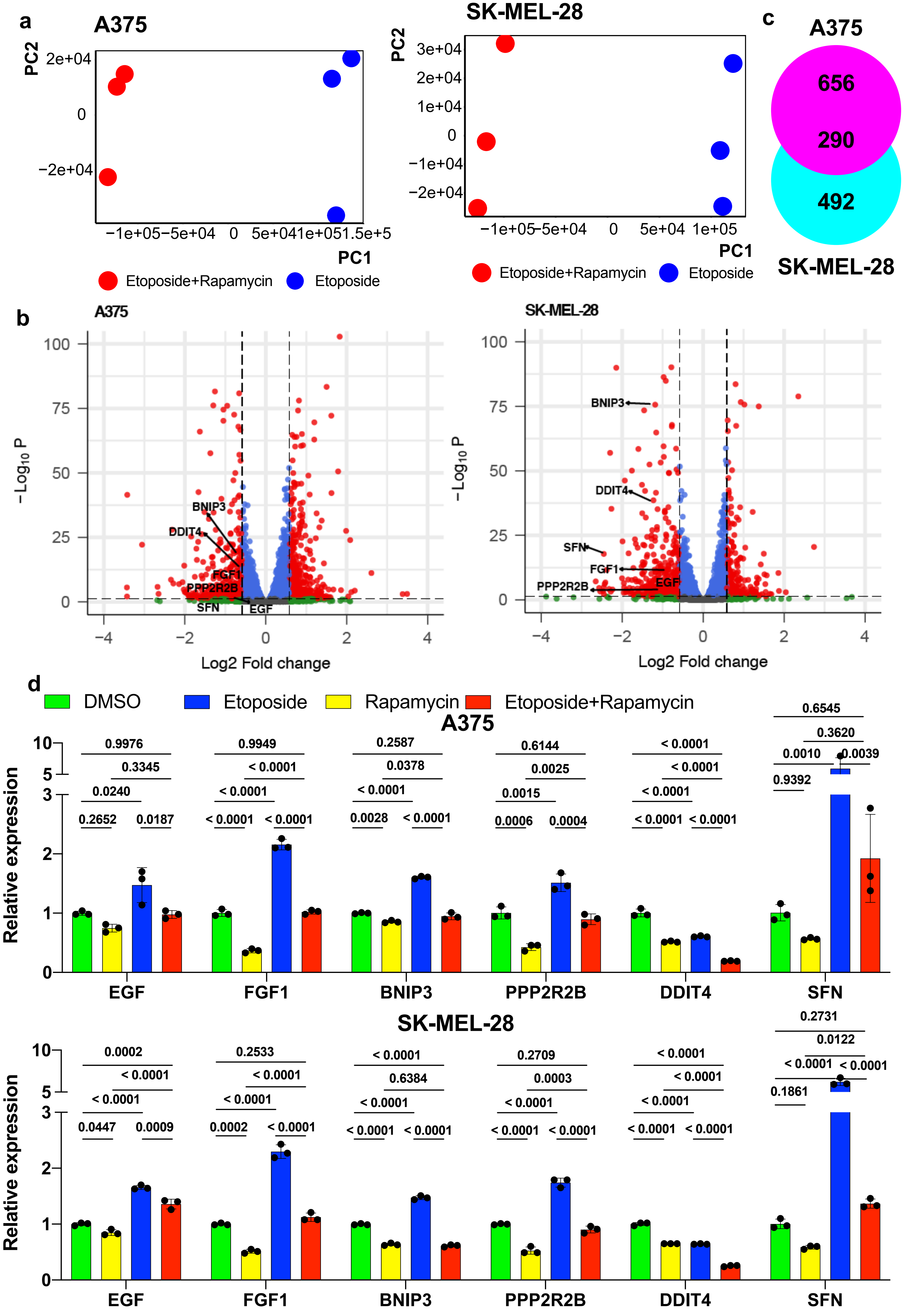

Supplementary Fig. 6 Analysis of autophagy sensitizing drug response. (a) PCA analysis of RNA-seq data from two groups in A375 and SK-MEL-28. (b) Volcano plot of differentially expressed genes for etoposide + rapamycin vs. etoposide in A375 and SK-MEL-28. (c) The venn plot of differentially expressed genes of etoposide + rapamycin vs. etoposide in A375 and -SK-MEL-28. (d) Relative expression of EGF, FGF1, BINP3, PPP2R2B, SFN and DDIT4 in four groups (n = 3) of A375 and SK-MEL-28 by RT-PCR. Data was presented as means ± SD. The difference in multiple groups was estimated by one-way ANOVA analysis. *P < 0.05; **P < 0.01; ***P < 0.001; ****P < 0.0001; ns is not significant.

Supplementary Fig. 7

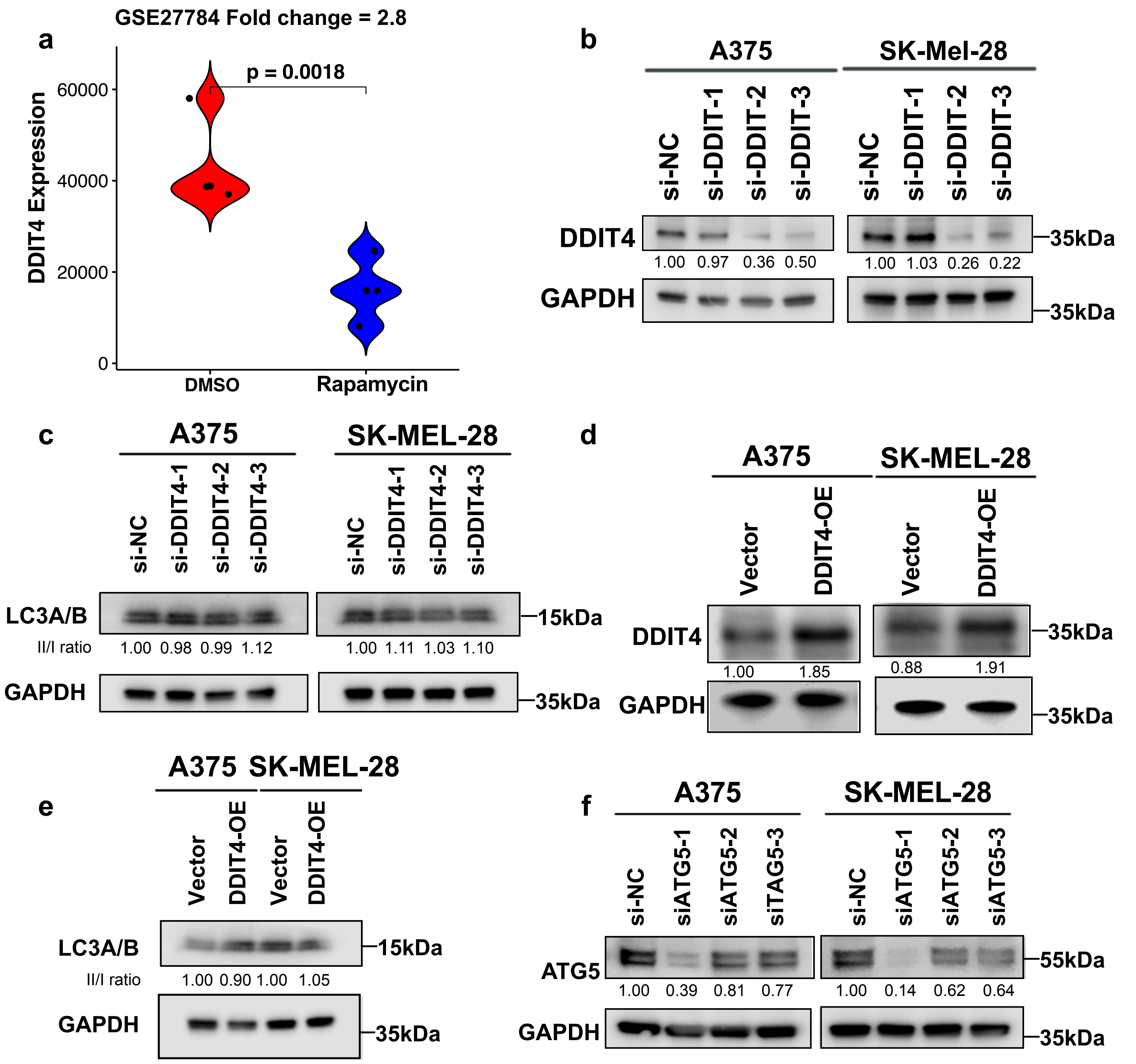

Supplementary Fig. 7 Expression of DDIT4 and the efficiency and specificity of DDIT4 siRNAs or DDIT4-OE lentivirus and ATG5 siRNAs in vitro. (a) The expression difference of DDIT4 between the DMSO group and the rapamycin group based on the public dataset, GSE27784. Two-sided student’ s t test was used for estimation of difference in two groups. (b) The western blot of DDIT4 in A375 and SK-MEL-28 cells transfected with si-NC or DDIT4 siRNAs. DDIT4 band intensities normalized to si-NC are displayed below the blots. (c) The autophagy status of A375 and SK-MEL-28 cells transfected with si-NC or DDIT4 siRNAs. The ratios of band intensities for the LC3-II/LC3-I normalized to si-NC are displayed below the blots. (d) The western blot of DDIT4 in A375 and SK-MEL-28 cells transfected with vector or DDIT4-OE lentivirus. DDIT4 band intensities normalized to Vector are displayed below the blots. (e) Western blot of autophagy markers in A375 and SK-MEL-28 cells transfected with vector or DDIT4-OE lentivirus. The ratios of band intensities for the LC3-II/LC3-I normalized to Vector are displayed below the blots. (f) The western blot of ATG5 in A375 and SK-MEL-28 cells transfected with si-NC or ATG5 siRNAs. ATG5 band intensities normalized to si-NC are displayed below the blots.(b-f) See Supplementary Fig. 8 for uncropped data. Similar results were observed in 3 (Supplementary Fig. 7b), 2 (Supplementary Fig. 7c), 3 (Supplementary Fig. 7d), 2 (Supplementary Fig. 7e), 3 (Supplementary Fig. 7f) independent experiments, respectively.

Supplementary Fig. 8

Fig. 7c

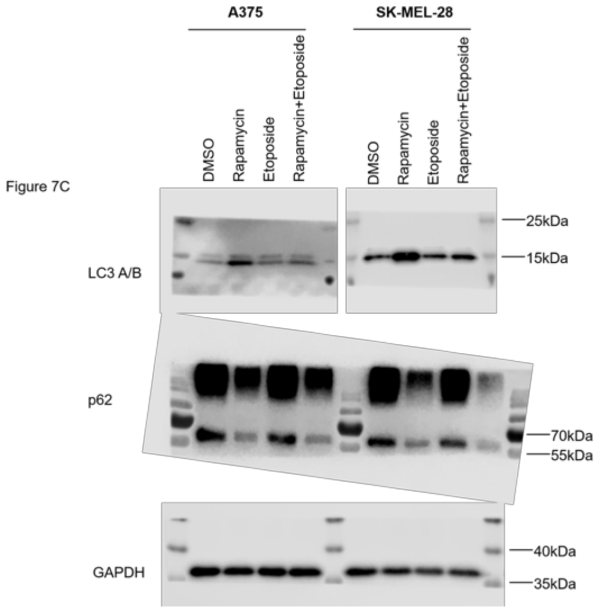

Fig. 7g

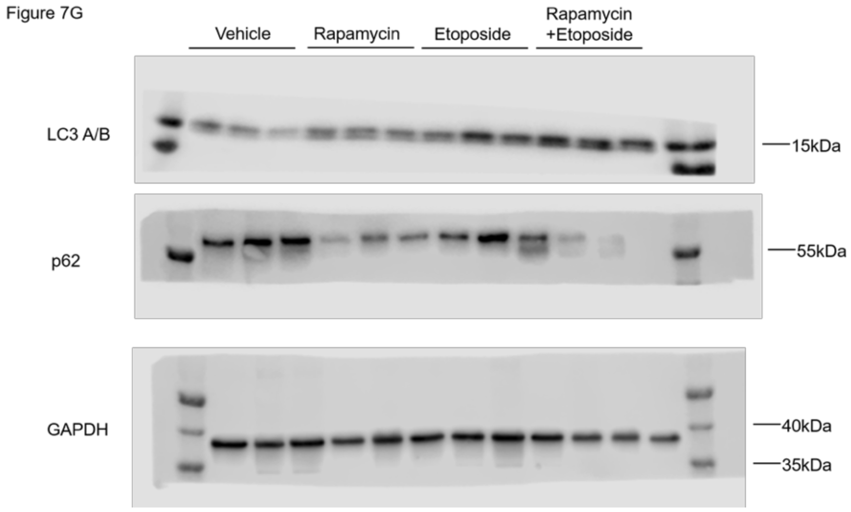

Fig. 8c

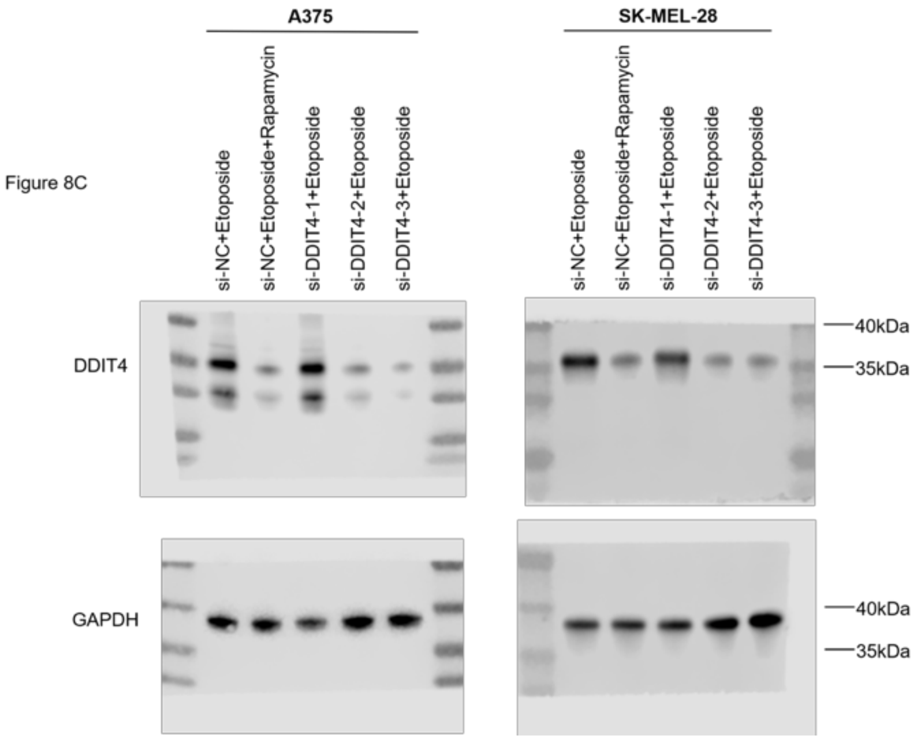

Fig. 8e

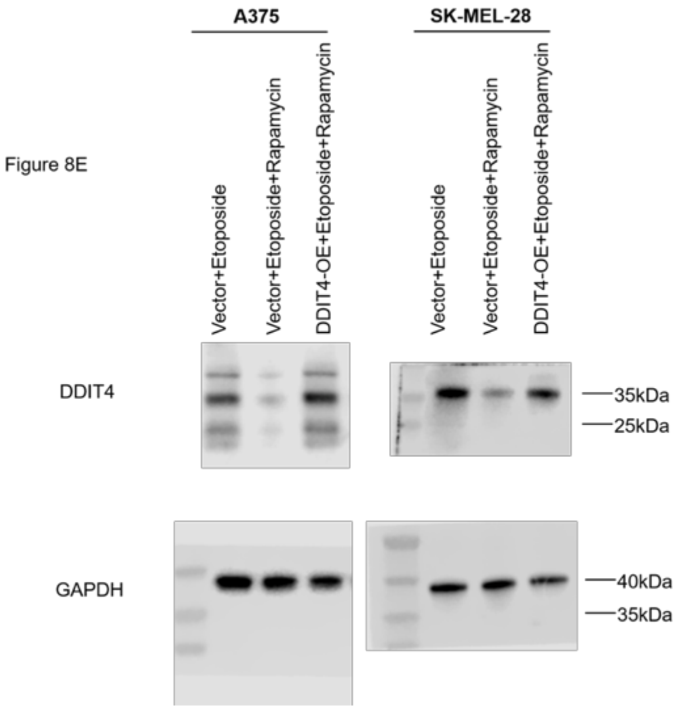

Fig. 8h

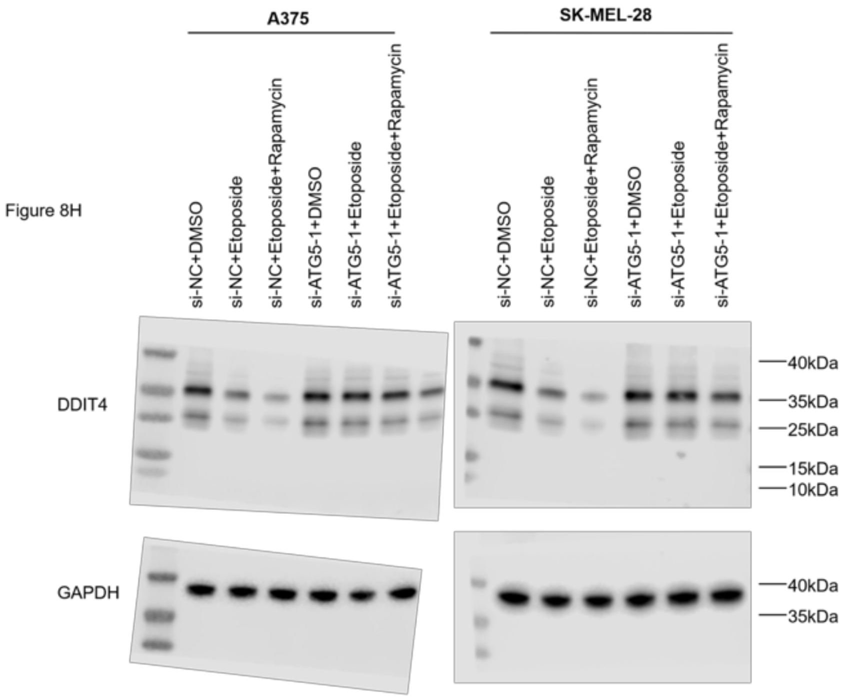

Supplementary Fig. 7b

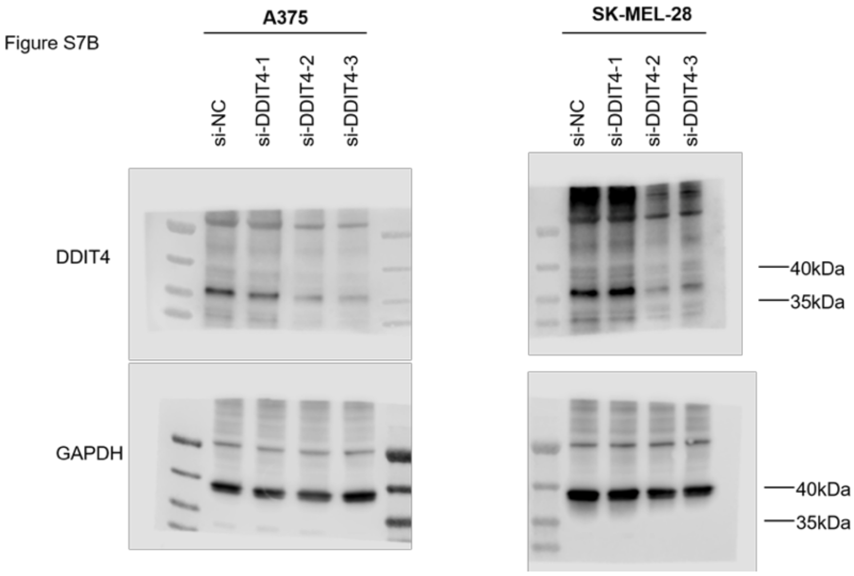

Supplementary Fig. 7c

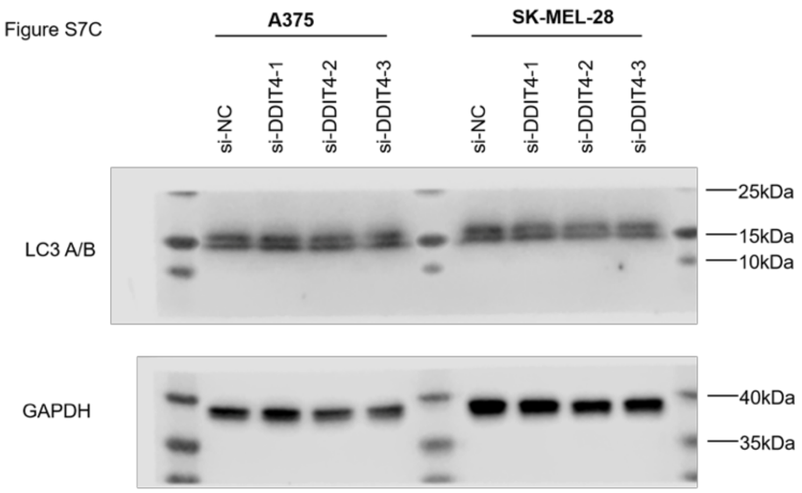

Supplementary Fig. 7d

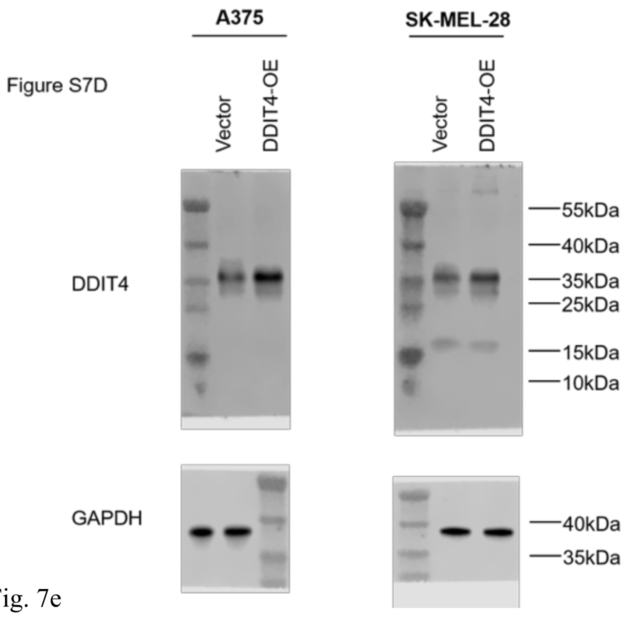

Supplementary Fig. 7e

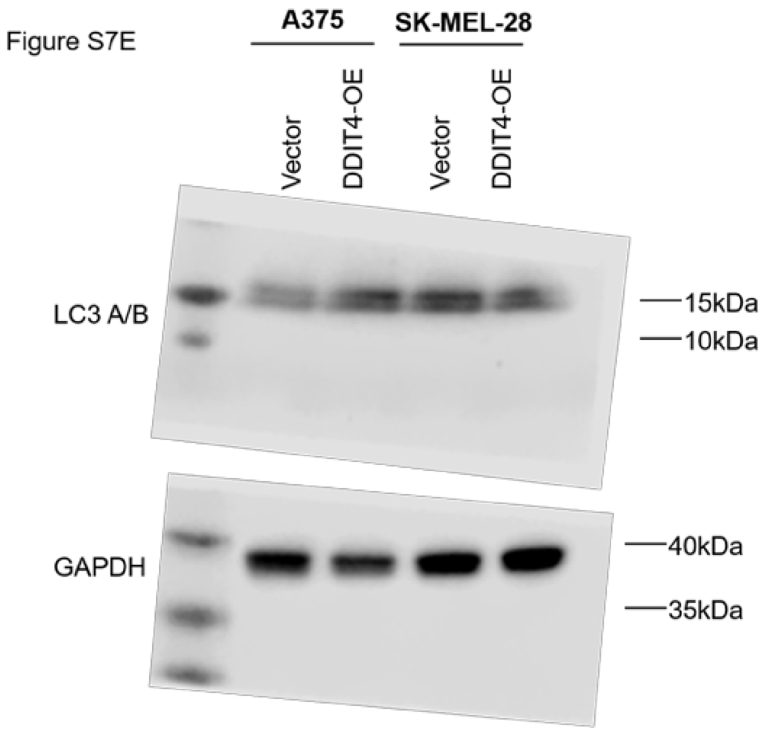

Supplementary Fig. 7f

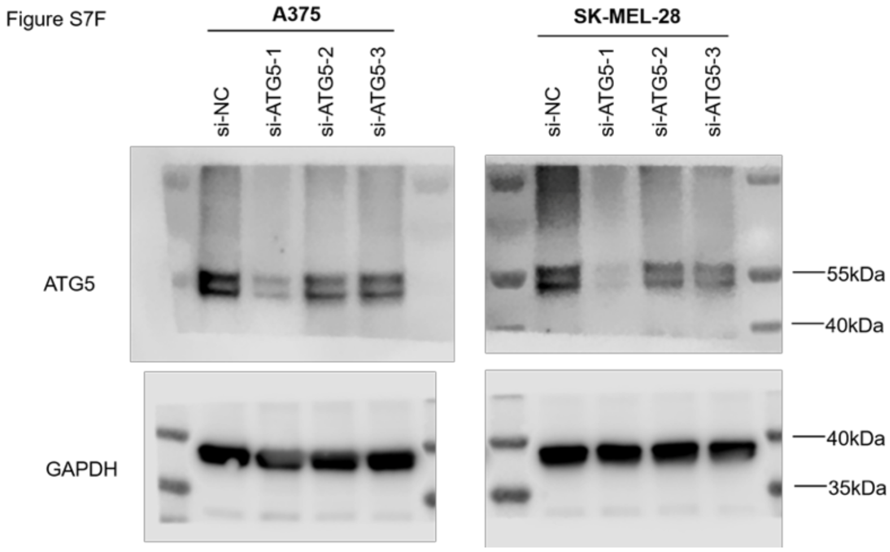

Supplement: Supplementary file 1 — Supplementary information [file 41467_2022_33946_MOESM1_ESM.pdf]
