# Supplementary material for: Multi-omics characterization of autophagy-related molecular features for therapeutic targeting of autophagy
Source: Nat Commun. 2022 Oct 26;13:6345. doi: 10.1038/s41467-022-33946-x (PMC9606020; doi:10.1038/s41467-022-33946-x)
Supplement: Supplementary file 3 — Description of Additional Supplementary Files [file 41467_2022_33946_MOESM3_ESM.pdf]

Description of Additional Supplementary Files

File Name: Supplementary Data 1
Description: Detailed information of the autophagy-associated 37-gene set.

File Name: Supplementary Data 2
Description: KEGG pathways with more than 10 significantly altered genes enriched in at least two cancer types. Enrichment of KEGG pathways was evaluated with 'clusterProfile' package.

File Name: Supplementary Data 3
Description: miRNA-target regulatory network and enriched pathways. Enrichment of KEGG pathways was evaluated with 'clusterProfile' package.

File Name: Supplementary Data 4
Description: TF-target regulatory network and enriched pathways. Enrichment of KEGG pathways was evaluated with 'clusterProfile' package.

File Name: Supplementary Data 5
Description: Sample information of RNA-seq.

File Name: Supplementary Data 6
Description: The overlapping differentially expressed genes of in etoposide + rapamycin vs. etoposide A375 and SK-MEL-28. The significance (p value) of differentially expressed genes was evaluated with 'DESeq2' package.

File Name: Supplementary Data 7
Description: The enriched cancer pathways of overlapping differentially expressed genes in A375 and SK-MEL-28. Enrichment of KEGG pathways was evaluated with 'clusterProfile' package.
