# Supplementary material for: Multi-omics characterization of autophagy-related molecular features for therapeutic targeting of autophagy
Source: Nat Commun. 2022 Oct 26;13:6345. doi: 10.1038/s41467-022-33946-x (PMC9606020; doi:10.1038/s41467-022-33946-x)
Supplement: Supplementary file 11 — Reporting Summary [file 41467_2022_33946_MOESM11_ESM.pdf]

Corresponding author(s): Leng Han

Last updated by author(s): Oct 6, 2022

Reporting Summary

Nature Portfolio wishes to improve the reproducibility of the work that we publish. This form provides structure for consistency and transparency in reporting. For further information on Nature Portfolio policies, see our Editorial Policies and the Editorial Policy Checklist.

Statistics

For all statistical analyses, confirm that the following items are present in the figure legend, table legend, main text, or Methods section.

n/a	Confirmed	
☐	☒	The exact sample size (*n*) for each experimental group/condition, given as a discrete number and unit of measurement
☐	☒	A statement on whether measurements were taken from distinct samples or whether the same sample was measured repeatedly
☐	☒	The statistical test(s) used AND whether they are one- or two-sided<br>*Only common tests should be described solely by name; describe more complex techniques in the Methods section.*
☐	☒	A description of all covariates tested
☐	☒	A description of any assumptions or corrections, such as tests of normality and adjustment for multiple comparisons
☐	☒	A full description of the statistical parameters including central tendency (e.g. means) or other basic estimates (e.g. regression coefficient) AND variation (e.g. standard deviation) or associated estimates of uncertainty (e.g. confidence intervals)
☐	☒	For null hypothesis testing, the test statistic (e.g. *F*, *t*, *r*) with confidence intervals, effect sizes, degrees of freedom and *P* value noted<br>*Give P values as exact values whenever suitable.*
☒	☐	For Bayesian analysis, information on the choice of priors and Markov chain Monte Carlo settings
☐	☒	For hierarchical and complex designs, identification of the appropriate level for tests and full reporting of outcomes
☐	☒	Estimates of effect sizes (e.g. Cohen's *d*, Pearson's *r*), indicating how they were calculated

Our web collection on statistics for biologists contains articles on many of the points above.

Software and code

Policy information about availability of computer code

Data collection	No software was used for data collection.
Data analysis	All data analysis was performed by R version 4.0.5, GraphPad Prism v 9.00 and Linux system.We performed KEGG pathway enrichment analysis by using the 'clusterProfiler' package; we calculated the ssGSEA score of samples from 6 datasets using the GSVA package. Protein bands were evaluate by Image J.

For manuscripts utilizing custom algorithms or software that are central to the research but not yet described in published literature, software must be made available to editors and reviewers. We strongly encourage code deposition in a community repository (e.g. GitHub). See the Nature Portfolio guidelines for submitting code & software for further information.

Data

Policy information about availability of data

All manuscripts must include a data availability statement. This statement should provide the following information, where applicable:

- Accession codes, unique identifiers, or web links for publicly available datasets
- A description of any restrictions on data availability
- For clinical datasets or third party data, please ensure that the statement adheres to our policy

The raw data of RNA-seq in this study were deposited in Sequence Read Archive (SRA, https://www.ncbi.nlm.nih.gov/sra/) under accession ID SRP341179 [https://www.ncbi.nlm.nih.gov/sra/?term=SRP341179]. The processed RNA-seq dataset were deposited in Gene Expression Omnibus (GEO, https://www.ncbi.nlm.nih.gov/geo/) under accession ID GSE185814 [https://www.ncbi.nlm.nih.gov/geo/query/acc.cgi?acc=GSE185814]. The public expression datasets were obtained from GEO, including GSE27784 [https://www.ncbi.nlm.nih.gov/geo/query/acc.cgi?acc=GSE27784], GSE107600 [https://www.ncbi.nlm.nih.gov/geo/query/acc.cgi?acc=GSE107600], GSE117189 [https://www.ncbi.nlm.nih.gov/geo/query/acc.cgi?acc=GSE117189], GSE129204 [https://www.ncbi.nlm.nih.gov/geo/query/acc.cgi?

acc=GSE129204], GSE106175 [https://www.ncbi.nlm.nih.gov/geo/query/acc.cgi?acc=GSE106175], GSE90444 [https://www.ncbi.nlm.nih.gov/geo/query/acc.cgi?acc=GSE90444], and GSE31397 [https://www.ncbi.nlm.nih.gov/geo/query/acc.cgi?acc=GSE31397]. The multi-omics datasets (mRNA expression, miRNA expression, protein expression, somatic mutations, and SCNA) and clinical data (age, gender, smoking, etc.) from 32 tumor types were downloaded from the TCGA data portal (https://portal.gdc.cancer.gov/). The tumor purity of TCGA samples was downloaded from Tumor IMmune Estimation Resource (http://cistrome.org/TIMER/download.html) and https://doi.org/10.5281/zenodo.253193 and integrated. The drug repurposing information with drug-target was downloaded from The Drug Repurposing Hub (https://clue.io/repurposing-app). Uncropped scans of all blots underlying Figs. 7c, 7g, 8c, 8e, 8h and Supplementary Figs. 7b-7f are provided as a Supplementary Fig. 8. Intensity values estimated by Image J are provided in Source data. Source data are provided with this paper.

Field-specific reporting

Please select the one below that is the best fit for your research. If you are not sure, read the appropriate sections before making your selection.

☒ Life sciences ☐ Behavioural & social sciences ☐ Ecological, evolutionary & environmental sciences

For a reference copy of the document with all sections, see nature.com/documents/nr-reporting-summary-flat.pdf

Life sciences study design

All studies must disclose on these points even when the disclosure is negative.

Sample size	Sample size of cell biology experiments and number of animals were chosen based on our previous experiences of carrying out similar experiments and published work. In this study, we analyzed all tumors with multi-omics data and clinical data from TCGA data portal (https://tcgadata.nci.nih.gov/tcga/) (n = 9686). We included 24 cancer types with ≥ 30 samples in both autophagy score-high and autophagy score-low groups for further analysis. Drug responses for 1,074 cancer cell lines were obtained from Drug Sensitivity in Cancer (GDSC, http://www.cancerrxgene.org/downloads). For drug response experiments, we used 3 biological replicates in vitro, and 7 biological replicates in vivo. RNA-seq with 3 biological replicates in case and control was performed, respectively. The relative mRNA expression was tested with 3 biological replicates for each group in vitro.
Data exclusions	We kept 24 cancer types with sample size ≥ 30 in both autophagy score-high and autophagy score-low groups for further analysis.
Replication	All data analyses and experimental findings are reproducible. Experiments were performed in at least 3 biologically independent replicates.
Randomization	Samples were allocated to groups based on autophagy status. For the xenograft model, mice were randomly allocated into four groups receiving different treatment.
Blinding	Investigator was blinded to the group assignment at the time of quantification.

Reporting for specific materials, systems and methods

We require information from authors about some types of materials, experimental systems and methods used in many studies. Here, indicate whether each material, system or method listed is relevant to your study. If you are not sure if a list item applies to your research, read the appropriate section before selecting a response.

Materials & experimental systems

n/a	Involved in the study	
☐	☒	Antibodies
☐	☒	Eukaryotic cell lines
☒	☐	Palaeontology and archaeology
☐	☒	Animals and other organisms
☒	☐	Human research participants
☒	☐	Clinical data
☒	☐	Dual use research of concern

Methods

n/a	Involved in the study	
☒	☐	ChIP-seq
☐	☒	Flow cytometry
☒	☐	MRI-based neuroimaging

Antibodies

Antibodies used	REDD1 Specific Rabbit polyAb: 1:1000 for WB, Proteintech, Cat. 10638-1-AP, REDD1 specific fusion protein Ag0965, Lot. 00086142; anti-GAPDH: 1:50000 for WB, Proteintech, Cat. 60004-1-Ig, GAPDH fusion protein Ag0766 Lot. 10008047; Atg5 (D5F5U) Rabbit mAb: 1:1000 for WB, Cell Signaling Technology, Cat. 12994T, Lot. 5; LC3A/B (D3U4C) Rabbit mAb: 1:1000 for WB, Cell Signaling Technology, Cat. 12741S, Lot. 5; SQSTM1/p62 (D5E2) Rabbit mAb: 1:1000 for WB, Cell Signaling Technology, Cat. 8025S, Lot. 6; HRP Goat Anti-Mouse IgG (H+L): 1:5000 for WB, ABclonal, Cat. AS003, Lot. 9300003001; HRP Goat Anti-Rabbit IgG (H+L): 1:5000 for WB, ABclonal, Cat. AS014. Lot. 9300014001.
Validation	REDD1 Specific Rabbit polyAb: IP, WB, ELISA. Species Specificity: human; Atg5 (D5F5U) Rabbit mAb: WB, IP. Species Specificity: human, mouse, rat; LC3A/B (D3U4C) Rabbit mAb: WB, IHC, IF, FCM. Species Specificity: human, mouse, rat; SQSTM1/p62 (D5E2) Rabbit mAb: WB, IP. Species Specificity: human, Monkey; anti-GAPDH: FC, IF, IP, WB, ELISA. Species Specificity: human, mouse, rat,

yeast, plant, zebrafish; HRP Goat Anti-Mouse IgG (H+L): WB, ELISA. WB: Mus musculus, Homo sapiens; HRP Goat Anti-Rabbit IgG (H+L): WB, ELISA. WB: Mus musculus, Homo sapiens.

Eukaryotic cell lines

Policy information about cell lines

Cell line source(s)	A375 , SK-MEL-28 and SK-MEL-5 cell lines were obtained from American Type Culture Collection (ATCC)
Authentication	The cell lines were directly obtained from ATCC and were not further authenticated.
Mycoplasma contamination	We confirmed that cell lines tested negative for mycoplasma contamination.
Commonly misidentified lines (See ICLAC register)	No commonly misidentified cell line was used in this study.

Animals and other organisms

Policy information about studies involving animals; ARRIVE guidelines recommended for reporting animal research

Laboratory animals	BALB/c nude mice, male, 4-week-old. Animals were housed under specific pathogen-free condition. The housing conditions were strictly following the ethical regulations (ambient temperature of 22-25°C; relative humidity of 50-60%; 12h/12h light/dark cycle; ad libitum access to food and water). The mice were randomly allocated into four groups (n = 7).
Wild animals	The study did not involve wild animals.
Field-collected samples	The study did not involve samples collected from the field.
Ethics oversight	All animal experiments were approved by the Animal Care and Use Committee of Central South University (Changsha, Hunan, China).

Note that full information on the approval of the study protocol must also be provided in the manuscript.

Flow Cytometry

Plots

Confirm that:

☒ The axis labels state the marker and fluorochrome used (e.g. CD4-FITC).

☒ The axis scales are clearly visible. Include numbers along axes only for bottom left plot of group (a 'group' is an analysis of identical markers).

☒ All plots are contour plots with outliers or pseudocolor plots.

☒ A numerical value for number of cells or percentage (with statistics) is provided.

Methodology

Sample preparation	A375 , SK-MEL-28 and SK-MEL-5 cell lines were obtained from American Type Culture Collection (ATCC)
Instrument	FACS LSR II Fortessa (BD Biosciences)
Software	FlowJo software (Tree Star)
Cell population abundance	About 20, 000 single cells per sample were sorted and analyzed.
Gating strategy	FSC-A and SSC-A to exclude cellular debris. FSC-A and FSC-H to exclude doublets. Gating strategies are shown in Supplementary Figure S5C.

☒ Tick this box to confirm that a figure exemplifying the gating strategy is provided in the Supplementary Information.
